# A psychological investigation of prospective teachers’ attitudes and motivations towards distance learning

**DOI:** 10.3389/fpsyg.2025.1579683

**Published:** 2025-11-25

**Authors:** İsmail Karakuş

**Affiliations:** Department of Basic Education, Faculty of Education, Mersin University, Mersin, Türkiye

**Keywords:** pre-service teacher, distance learning, attitude, motivation, digital learning environments

## Abstract

**Introduction:**

This study aims to examine the psychological aspects of pre-service teachers’ attitudes and motivation towards distance learning and the relationship between these variables. Psychological constructs such as self-efficacy, intrinsic motivation, and learning engagement were considered key factors in understanding these relationships.

**Methods:**

The study was conducted with 388 volunteer pre-service teachers enrolled in a state university in Türkiye during the 2023–2024 academic year, selected through stratified sampling to represent all departments.

**Results:**

Correlation and regression analyses revealed that both attitudes and motivation towards distance learning were slightly above the moderate level. A significant and positive relationship was found between attitudes and motivation (*r* = 0.65, *p* < 0.01), with attitudes explaining approximately 43% of the variance in motivation (R^2^ = 0.43).

**Discussion:**

These findings suggest that psychological factors—such as perceived competence and digital learning adaptability—play a crucial role in shaping motivation. The results highlight the importance of integrating psychological support mechanisms into distance education to enhance motivation and engagement among pre-service teachers.

## Introduction

Epidemics, natural disasters, or similar extraordinary events can make distance learning an important alternative for countries or institutions. From a psychological perspective, such crises can lead to increased anxiety, cognitive overload, and difficulties in adapting to new learning environments (Bao, 2020). Following the recent earthquakes in Türkiye, universities moved instruction online to ensure educational continuity. This decision aimed to meet students’ learning needs.

Natural disasters, such as earthquakes, pose not only infrastructural and technological challenges but also significant psychological burdens on students and educators (Chaturvedi et al., 2021). Earthquakes can seriously damage the physical structure of schools and prevent students from accessing a safe educational environment. In such cases, educational institutions are forced to turn to distance education methods. However, limited internet access and weak technological infrastructure can hinder this transition. More importantly, traumatic experiences may reduce motivation, increase cognitive load, and impair learning efficiency, thereby weakening engagement with distance education (Brooks et al., 2020). While previous research has emphasized the roles of trauma, anxiety, and psychological resilience following natural disasters (Brooks et al., 2020; Chaturvedi et al., 2021), the current study focuses specifically on students’ attitudes and motivation towards distance learning rather than measuring these variables directly.

While families with poor financial conditions struggle to access distance education re-sources, psychosocial disparities can also widen, leading to increased academic stress and lower self-efficacy among disadvantaged students (Mishra et al., 2020). Thus, distance learning can help overcome physical, infrastructural, and financial barriers. Nevertheless, success in digital environments also hinges on psychological factors such as resilience, self-regulation, and perceived control (Almazova et al., 2020).

Among these psychological factors, two constructs are particularly important in understanding students’ adjustment to distance learning: self-efficacy and cognitive adaptability. Self-efficacy refers to individuals’ beliefs in their ability to organize and execute the actions required to manage prospective situations (Bandura, 1997). Students with higher self-efficacy perceive themselves as capable of overcoming technical difficulties, managing digital tools, and adapting to novel learning environments (Zimmerman, 2000). Numerous studies have emphasized that self-efficacy is positively associated with academic performance, persistence, and motivation in online education settings (Artino, 2008; Lee et al., 2014). Moreover, self-efficacy mediates the relationship between perceived challenges and learning outcomes by enhancing students’ confidence in problem-solving (Schunk and DiBenedetto, 2020).

Cognitive adaptability, on the other hand, refers to individuals’ ability to adjust their cognitive and behavioral strategies in response to new, changing, or uncertain learning conditions (Martin et al., 2013; Haynie, 2005). Students with higher cognitive adaptability are more likely to modify their learning approaches to accommodate changes in course delivery, technological platforms, or instructional demands (Ployhart and Bliese, 2006). In distance learning, where rapid transitions and technological variations are frequent, cognitive adaptability enables learners to manage novelty, uncertainty, and ambiguity more effectively (Toland and Carrigan, 2011). These adaptive skills are particularly valuable in emergency-based online learning contexts, such as those necessitated by natural disasters, where flexibility and problem-solving are crucial for academic success (Bozkurt and Sharma, 2020).

In combination, both self-efficacy and cognitive adaptability contribute to students’ motivation, engagement, and overall psychological readiness for distance learning (Ryan and Deci, 2020; Schunk and DiBenedetto, 2020). Without addressing these underlying psychological resources, students may experience difficulties in maintaining focus, overcoming challenges, and sustaining academic performance during prolonged periods of online education.

Although the theoretical framework discussed above encompasses multiple psychological constructs such as self-efficacy, cognitive adaptability, and resilience, these variables were addressed conceptually to provide a broader psychological context. The present study empirically focuses on pre-service teachers’ attitudes and motivation toward distance learning, which are considered key observable indicators of psychological readiness in digital education environments. In this sense, attitude can be viewed as a proximal behavioral reflection of self-efficacy and adaptability, demonstrating how underlying psychological resources manifest in students’ engagement with online learning.

### Distance learning

Distance learning is a multidisciplinary field that has evolved over time and meets the diverse needs of learners. However, in emergency contexts, such as during natural disasters or pandemics, the psychological readiness of students and teachers becomes a key determinant of distance learning success (Bozkurt and Sharma, 2020; Hodges et al., 2020). Rather than creating a permanent and robust educational ecosystem, emergency distance learning primarily aims to provide rapid and reliable access to education (Hodges et al., 2020). From a cognitive psychology perspective, the shift to distance learning requires students to develop new self-regulatory strategies, sustain attention in virtual environments, and cope with the lack of direct social interaction (Schunk and DiBenedetto, 2020). Research highlights that motivation, engagement, and perceived ease of use are critical for the effectiveness of online learning platforms (Hjeltnes and Hansson, 2005; Kohnke, 2021).

Uluğ and Kaya (1997) define distance learning as ‘all educational practices structured in environments where teachers and students are separated in time and space.’ The United States Distance Learning Association describes it as ‘the acquisition of knowledge and skills through mediated information and instruction’ (Roblyer and Edwards, 2000). Moore and Kearsley (2005) define it as planned teaching and learning conducted in separate locations via electronic communication. Compared with traditional instruction, distance learning offers a systematic technological structure (Aguilera-Hermida, 2020), flexibility and comfort (Fatonia et al., 2020), and instruction unconstrained by time and place (Yates et al., 2020).

A functional distance learning process requires attention to several issues. Institutions should address inadequate digital infrastructure and technical problems (Dutta and Smita, 2020), train teachers in digital competencies, and ensure economic resources for devices and internet access (Barakabitze et al., 2019; Mtebe and Raisamo, 2014).

Tulinayo et al. (2018) reported low technological competence among teachers in developing countries and reluctance to adopt new solutions. Palvia et al. (2018) noted that students feel lonelier and more isolated in distance learning than in face-to-face education. Coman et al. (2020) further highlighted barriers such as limited interaction and delayed feedback. Paying attention to such variables in distance learning and developing appropriate strategies aimed at removing the barriers associated with online learning and organizing the learning process accordingly can affect individuals’ attitudes and motivation (Adarkwah, 2020; Tapalova and Myrzalieva, 2017).

### Attitude for distance learning

Attitudes towards distance learning are shaped by multiple psychological dimensions, including cognitive beliefs, affective responses, and prior experiences with digital education (Ajzen, 2011). Student attitudes towards distance learning can provide information about the quality of distance education use (Liaw et al., 2007). Effective implementation of distance learning is associated with users developing a positive psychological disposition, including adaptability and openness to technology-driven learning environments (Takir, 2022).

According to Tavşancıl (2002), attitude is a phenomenon that is acquired through learning, directs the behavior of the individual and causes bias in the decision-making process. In the context of digital learning, attitude formation is closely linked to self-efficacy, perceived control over learning, and prior exposure to online education (Bandura, 1997). Studies have shown that students who perceive distance learning as flexible and beneficial tend to develop higher engagement levels and experience lower cognitive resistance (Yenilmez et al., 2017). Determining the thoughts, approaches and attitudes of individuals about these processes is important for the successful and efficient operation of the system (Şahin, 2007; Altan and Seferoğlu, 2009). An important point of the distance learning process is students’ motivation.

### Motivation for distance learning

Motivation is a key psychological factor in distance learning, influencing persistence, engagement, and overall academic performance (Ryan and Deci, 2021). In this context, motivation is generally classified into two main categories: intrinsic motivation and extrinsic motivation. Intrinsic motivation refers to the drive to engage in learning activities for inherent satisfaction, such as curiosity, interest, enjoyment, or mastery goals. In contrast, extrinsic motivation involves engagement in tasks due to external rewards or pressures, such as grades, certificates, social recognition, or institutional requirements (Çetin and Kırbulut, 2006). Lepper (1988) defined motivation for learning as “an impulse fueled by a need, expectation, goal, and emotion.” In the context of distance education, maintaining motivation is particularly challenging due to factors such as social isolation, lack of immediate feedback, and distractions in the home environment (Zhou and Zhang, 2023). These challenges often reduce students’ sense of relatedness and autonomy—two key components of motivation according to the Self-Determination Theory (Ryan and Deci, 2021).

Olowo et al. (2020) argue that motivation is important in distance learning, Moore (1993) also stated that student motivation should be supported. Because, considering that there is a relationship between learning motivation and overall satisfaction with the courses (Bailey et al., 2021) and a positive relationship between students’ engagement, motivation and the level of specialization in professional skills (Rajabalee and Santally, 2021), it is necessary to ensure student motivation in distance learning processes.

In the present study, motivation is operationalized as a multidimensional construct incorporating both intrinsic and extrinsic components, as assessed by the second section of the “Motivation Questionnaire for E-learning Environments” (Kim, 2005; Yıldırım, 2012). This approach allows for the examination of students’ psychological engagement with online learning, focusing on their interest, persistence, perceived relevance, and response to external demands. Clarifying this distinction is important to understand which motivational mechanisms are most effective in promoting sustained learning behaviors in digital contexts.

### The relevance of the research

When the literature is examined, the attitudes of university students towards distance learning (Smidt et al., 2016; Bayram et al., 2019; Cansu, 2021; Halitoğlu, 2021; Marjerison et al., 2020; Kaban, 2021; Karagözoğlu and Gezer, 2022; Takir, 2022; Yazgan, 2022; Kaya et al., 2022) and motivation (Bertiz and Karoğlu, 2020; Malinauskas and Pozeriene, 2020; Özüdoğru, 2021). In the literature, the concepts of attitude and motivation have been studied separately or with different concepts. However, research focusing on the interplay between psychological constructs—such as attitudes, motivation, and self-efficacy—remains limited. Moreover, in this study, it is thought that examining the attitudes and motivations of university students towards distance learning and the relationship between them can contribute to the literature because it makes the study different and important. In particular, examining distance learning through the lens of psychological resilience, cognitive adaptation, and emotional regulation in the aftermath of a natural disaster may offer valuable insights for educational stakeholders. Understanding how students psychologically respond to sudden transitions to online education can contribute to the design of more effective distance learning policies and support mechanisms (Sicorello and Schmahl, 2021).

In addition, it is thought that examining the distance learning process, students’ attitudes and motivations by addressing a current problem due to a natural disaster (earth-quake) that may affect the emotional and psychological states of educators and students may be important for all stakeholders of education and may contribute to the literature. This study aims to examine the attitudes and motivations of university students towards distance learning and whether they differ according to various psychological and demo-graphic variables. The research specifically seeks to answer the following questions: In line with this purpose, in this study, university students; (1) what level are their attitudes? (2) Do their attitudes differ according to sex and grade level? (3) What is their level of motivation? (4) Do their motivations differ according to sex and grade level? (5) What is the relationship between their attitudes and motivation? (6) Do their attitudes predict their motivation? answers to such problems have been sought.

In line with the research aim and literature framework, the current study incorporates both exploratory and confirmatory dimensions. The exploratory aspect focuses on identifying pre-service teachers’ general attitudes and motivation levels towards distance learning and examining differences based on demographic variables. The confirmatory component tests specific statistical hypotheses regarding the predictive relationship between attitudes and motivation. The hypotheses tested in this study are as follows:

- H₀₁: There is no significant relationship between pre-service teachers’ attitudes towards distance learning and their motivation.
- H₁₁: There is a significant and positive relationship between pre-service teachers’ attitudes towards distance learning and their motivation.
- H₀₂: Pre-service teachers’ attitudes do not significantly predict their motivation towards distance learning.
- H₁₂: Pre-service teachers’ attitudes significantly predict their motivation towards distance learning.

## Materials and methods

This study analyses university students’ attitudes and motivation towards distance learning and the relationship between the two variables. The study was conducted based on the relational survey model. In line with this purpose, the study adopted a cross-sectional and exploratory correlational design, aiming to identify patterns rather than causal relationships between variables. Accordingly, the analyses were conducted to explore the predictive association between attitude and motivation, without testing mediation or moderation effects. Relational research is an analysis technique in which variables are related to each other and information is systematically integrated as theories begin to develop (Cohen et al., 2007). There are three things to look at in every relationship analysis. These are; whether there is a relationship, the meaning of the relationship, the direction of the relationship and the level of the relationship (Karasar, 2018). At the same time, statistical techniques such as correlation and regression are used to analyze the relationship between the variables measured in such studies. Based on one variable, the other variable can be predicted (Fraenkel and Wallen, 2006).

### Working group

The population of the study consists of the students of a state university studying distance education in the autumn term of the 2023–2024 academic year. In sample selection, stratified sampling, one of the probability-based sampling methods, was used to see the reflection of all departments in the research. Stratified sampling is used under the condition that subgroups exist in a universe with certain boundaries, and the point to be considered in this sampling model is to work on the universe based on the existence of subgroups within the universe rather than accepting the universe as a pure and similar phenomenon in itself (Yıldırım and Şimşek, 2013). In this study, stratification was performed based on students’ academic grade level and program type (undergraduate or pedagogical formation). Within each stratum, simple random sampling was used to ensure proportional representation of subgroups according to their size in the total student population. This approach was intended to increase the representativeness of the sample and ensure balanced participation from different student profiles. Random sampling method was applied for each subgroup and a sample of 388 participants was obtained. This sample size provides sufficient statistical power at 95% confidence level in the analyses based on the moderate effect size (*r* = 0.3) recommended by Cohen (1988). In the study, a sample size that meets the assumptions of the statistical analyzes used (such as correlation, regression and ANOVA) was reached. Calculations made with the G*Power analysis tool show that this sample size is sufficient to determine a moderate effect size (Faul et al., 2009). For regression analysis, a sample of 388 people can allow us to build a strong model explaining 43% of the dependent variable. Since the data was collected from a single university, the results of the study are specific to this region. They may not be generalizable to other regions, countries and continents. This can be considered as a limitation of the study. In this study, data were collected by ensuring the participation of all classes and branches. In order to conduct the study, the necessary permissions were obtained from Mersin University Ethics Committee Commission.

Of the pre-service teachers participating in the study, 287 (74%) were female and 101 (26%) were male. The sex of the participants was reported according to their self-reports in the personal information form according to their biological characteristics. 68 of them (17.5%) were 1st grade, 102 of them (26.3%) were 2nd grade, 23 of them (5.9%) were 3rd grade, 71 of them (18.3%) were 4th grade, 47 (12.1%) were graduated formation students and 77 (19.8%) were formation students continuing their undergraduate education.

### Data collection tools

‘Personal Information Form’, ‘Attitude Scale towards Distance Education’ and ‘Motivation Questionnaire for E-Learning Environments’ were used as data collection tools in the study.

In this study, self-report instruments were selected due to their practicality in collecting data from a large sample within a limited time frame, and their suitability for assessing subjective psychological constructs such as attitudes and motivation. These tools allow participants to reflect on their own perceptions and experiences, which are central to examining personal factors related to distance learning (Schunk and DiBenedetto, 2020).

### Personal information form

The personal information form was prepared by the researcher in line with the literature. In the form, there are survey questions prepared to determine the sex and class in-formation of the participant.

### Attitude scale towards distance education

The ‘Attitude Towards Distance Education Scale’ developed by Arslan (2021) con-sists of 5 factors and 21 items: satisfaction with the opportunities offered by the university in distance education, attitude towards lecturers, attitude towards online exams, communication and access in distance education, and comparison of distance education and face-to-face education. The scale is graded as “strongly disagree, disagree, undecided, agree and strongly agree.” The KMO value of the scale was 0.883 and Barlett’s test value was found to be significant. The total variance of the 5-factor structure was found to be 63.333%. Item factor loadings were found to be between 0.511 and 0.845. As a result of CFA, the fit indices were calculated within the acceptable range. The item analysis based on the lower and upper groups for item discrimination index was found to be significant (*p* < 0.05). Cronbach Alpha value, which is an indicator of internal consistency, was found to be 0.884 for the whole original scale and 0.896–0.658 for sub-factors. Based on the study group of this research, 0.92 was found for the whole scale and 0.92, 0.90, 0.86, 0.86, 0.78 and 0.73 for the sub-factors, respectively.

The 5-point Likert format was preferred because it allows for the measurement of attitudinal tendencies as continuous psychological constructs, enabling participants to express varying degrees of agreement and improving response sensitivity. The reliability coefficients indicate that the scale and its subdimensions provide satisfactory internal consistency for research purposes.

### Motivation questionnaire for e-learning environments

The “Motivation Questionnaire for E-learning Environments” developed by Kim (2005) was adapted into Turkish by Yıldırım (2012) and structured to consist of four sections. In this study, the second section of the questionnaire, which focuses on motivational components, was utilized. The original scale, which is based on a five-point Likert format (strongly disagree, disagree, neutral, agree, strongly agree), had a reported reliability coefficient of 0.70. In the present study, the reliability coefficient was found to be 0.83.

The use of a 5-point Likert scale was chosen for its appropriateness in capturing motivational intensity and variation among participants. The obtained reliability coefficient (*α* = 0.83) demonstrates acceptable internal consistency, confirming that the instrument is psychometrically suitable for measuring pre-service teachers’ motivation in digital learning environments.

Regarding the motivation scale, only the second section of the “Motivation Questionnaire for E-learning Environments” was used in this study. This section was selected because it specifically focuses on motivational components directly relevant to the research objectives. Limiting the scope to this section allowed for a more targeted assessment of students’ motivation while also minimizing respondent fatigue and improving data quality, following prior studies that have similarly used partial scales for specific constructs (Yıldırım, 2012).

Although the scale does not categorize motivation explicitly as intrinsic or extrinsic, its subdimensions reflect both types of motivational orientations. Specifically, the components of interest, confidence, and satisfaction are closely aligned with intrinsic motivation, as they relate to learners’ internal engagement, perceived competence, and personal fulfillment in the learning process. On the other hand, the relevance dimension corresponds to extrinsic motivation, as it captures the perceived usefulness and goal-related value of learning tasks, often influenced by external expectations or rewards. Thus, the scale allows for a multidimensional assessment of students’ motivation within both internal and external domains, consistent with the ARCS model developed by Keller (1987).

### Data collection process

Before data collection, all students participating in the study were informed about the purpose of the study with a comprehensive explanation. It was specifically stated that the results of the research would not affect school grades and that students’ names would not be collected. Data collection tools were collected online from the students via Google Form in the classroom environment. Participation in the study was voluntary. Informed consent was obtained from the participants before starting the study. It took an average of 15 min to complete the scales. The forms used in obtaining the research data were ad-ministered by the researcher personally.

### Analyzing the data

SPSS 25 program was used for data analysis. Before conducting simple linear regression analyses, certain conditions must be met. These conditions increase the validity and reliability of the model. Missing data control, outlier detection, linearity and normality tests, which are the basic conditions that must be met for simple linear regression, were performed. Missing data were checked before the analysis and no missing data were found. Outliers were checked for independent variables. In independent variables, z scores were calculated for univariate outliers and Mahalanobis distance values were calculated for multivariate outliers. While z standard values outside the range of +3.29 to +3.29 are referred to as univariate outliers, Mahalanobis distance values with probability less than *p* = 0.0001 are referred to as multivariate outliers (Tabachnick and Fidell, 2007). As a result, when the z standard values and Mahalanobis distance values are analyzed, no participant was detected above the critical value in the chi- square table. Skewness and kurtosis coefficients were calculated to determine the conformity of the data to normal distribution. Since the calculated kurtosis and skewness coefficients were between −1.5 and +1.5, it was accepted that the data were normally distributed (Tabachnick and Fidell, 2007). For the line-arity assumption, there should be a linear relationship between the independent variable (X) and the dependent variable (Y; Cohen et al., 2013). When the scatter plot was analyzed, it was assumed to be linear.

In this study, independent sample *t*-test was used to examine the difference in the attitudes and motivations of prospective teachers towards distance learning according to the gender variable. Analysis of variance (ANOVA) was used to examine the difference in the grade level variable.

After one-way ANOVA, LSD test was used to determine significant differences be-tween two groups when the variances were equal, and Games Howell test was used in findings that violated homogeneity. The effect size of the difference was calculated and interpreted according to Cohen (2013). Arithmetic mean and standard deviation values were calculated to determine the participants’ perceptions of the variables. In this study, Pearson correlation coefficients were calculated to examine the relationship between teacher candidates’ attitudes and motivation towards distance learning. In this study, regression analysis was conducted to examine to what extent the attitudes of prospective teachers towards distance learning predict motivation. The significance level of the study was taken as 𝛼=0.05.

Simple linear regression is used to model and analyze the linear relationship be-tween a dependent variable and an independent variable. This method is used to examine the effect of a single independent variable on the dependent variable and to predict the possible value of the independent variable and the dependent variable (Cohen et al., 2013). Simple linear regression is used to visualize and understand the linear relationship between two variables. The regression line shows the linearity of the relationship between the data and analyzes whether this relationship is strong or weak (Field, 2018). It is also used to test whether a particular independent variable has a significant effect on the de-pendent variable. This helps to test hypotheses and evaluate the relationships between variables in research. Therefore, simple linear regression analysis was preferred in this study.

## Results

Within the scope of the research, the level of pre-service teachers’ attitudes and level of motivation, whether they differ according to some variables and the relationship be-tween these two variables and the findings on the level of attitude predicting motivation are presented.

### Findings on pre-service teachers’ attitudes towards distance learning

In the study, the mean and standard deviation values of students’ attitudes towards distance learning were analyzed. “In the study, the mean attitude score of students towards distance learning was 3.07 (SD = 0.699), which can be considered slightly above the middle level. This indicates that students’ attitudes generally show a trend from neutral to positive, but they do not exhibit a distinctly strong positive attitude.

To determine whether students’ attitudes changed according to sex, an independent samples t-test was conducted. The analysis showed that female students (𝑥̅ = 3.04, SD = 0.697) and male students (𝑥̅ = 3.15, SD = 0.699) reported similar attitude levels, and this difference was not statistically significant (t(386) = −1.42, *p* = 0.155).

The status of students’ attitudes according to their classes is presented in Table 1.

**Table 1:** ANOVA findings to determine the attitudes of pre-service teachers’ towards distance learning according to their grade levels.

Variables	Grade levels	N	$\bar{X}$	Ss	F	*p*	Eta squared (𝜂^2^)
Attitude	1	68	3.30	0.495	14.281	0.000	0.1574
	2	102	2.86	0.594			
	3	23	2.66	0.899			
	4	71	2.78	0.826			
	Formation (Graduate)	47	3.15	0.536			
	Formation (Undergraduate)	77	3.46	0.611			
	Total	388	3.07	0.699			
Satisfaction with the facilities offered by the university	1	68	3.35	0.247	7.896	0.000	0.0674
	2	102	2.90	0.352			
	3	23	2.60	0.268			
	4	71	2.75	0.278			
	Formation (Graduate)	47	3.44	0.336			
	Formation (Undergraduate)	77	3.18	0.296			
	Total	388	3.04	0.288			
Attitude towards faculty members	1	68	3.37	0.302	8.997	0.000	0.1125
	2	102	2.96	0.244			
	3	23	2.56	0.163			
	4	71	2.64	0.350			
	Formation (Graduate)	47	3.80	0.240			
	Formation (Undergraduate)	77	3.04	0.115			
	Total	388	3.07	0.243			
Attitude towards online exams	1	68	3.42	0.250	11.458	0.000	0.0982
	2	102	2.66	0.291			
	3	23	2.88	0.283			
	4	71	2.95	0.158			
	Formation (Graduate)	47	3.22	0.302			
	Formation (Undergraduate)	77	3.25	0.277			
	Total	388	3.08	0.268			
Communication and access	1	68	3.41	0.355	10.256	0.000	0.1624
	2	102	2.89	0.248			
	3	23	2.57	0.353			
	4	71	2.77	0.224			
	Formation (Graduate)	47	3.32	0.286			
	Formation (Undergraduate)	77	3.34	0.302			
	Total	388	3.05	0.284			
Comparison of distance learning and face-to-face education	1	68	3.38	0.345	6.658	0.000	0.1578
	2	102	2.78	0.304			
	3	23	3.31	0.256			
	4	71	3.24	0.298			
	Formation (Graduate)	47	3.42	0.286			
	Formation (Undergraduate)	77	3.03	0.312			
	Total	388	3.19	0.331			

As shown in Table 1, the attitudes of pre-service teachers towards distance education were examined in relation to their grade levels to determine whether statistically significant differences exist. The Attitude Scale towards Distance Education employed in this research consists of five sub-factors: (1) satisfaction with university opportunities in distance education, (2) attitudes towards instructors in distance education, (3) attitudes towards online examinations, (4) communication and access in distance education, and (5) comparison of distance education and face-to-face learning. According to the ANOVA results, significant differences were found across both the total attitude scores and each of the sub-factors depending on the students’ grade levels.

The analysis revealed that that the attitude levels of the students differed statistically according to the grades (*F* = 14.281; *p* < 0.05). In order to determine the difference in the attitude levels of the students according to the grades, LSD test was performed due to the homogeneous distribution of the groups. According to the results of the analysis, there is a difference between 1st and 2nd, 3rd and 4th grades in favor of 1st grade; between 2nd grade and formation (graduate) in favor of formation (graduate) and between 2nd grade and formation (under-graduate) in favor of formation (undergraduate). There are significant differences between 3rd grade and formation (undergraduate) in favor of formation; between 4th grade and formation (undergraduate) in favor of formation (undergraduate) and between 4th grade and formation (graduate) in favor of formation (graduate). There are significant differences between formation (undergraduate) and formation (graduate) in favor of formation (undergraduate). This finding shows that grade levels and educational programs have different effects on students’ attitudes. Formation (undergraduate) and (graduate) programs may shape their attitudes as they offer students more educational content and experiences. Also, 1st year students usually start university life with higher motivation, which may lead to a decline in their attitudes in the following years.

Regarding the first sub-factor, “satisfaction with university opportunities,” first-year students reported significantly higher mean scores compared to second-, third-, fourth-year students, and formation graduates. (*F*(5, 382) = 7.896, *p* < 0.001, η^2^ = 0.0674). In addition, undergraduate formation students had significantly higher scores than third- and fourth-year students (F(5, 382) = 7.896, *p* < 0.001, η^2^ = 0.0674). Concerning the second sub-factor, “attitudes towards instructors,” first-year students again exhibited more favorable attitudes than third- and fourth-year students F(5, 382) = 8.997, *p* < 0.001, η^2^ = 0.1125), while formation graduates scored significantly higher than fourth-year students. Furthermore, undergraduate formation students showed significantly more positive attitudes compared to second-, third-, and fourth-year students (F(5, 382) = 8.997, *p* < 0.001, η^2^ = 0.1125).

In the third sub-factor, “attitudes towards online examinations,” first-year students differed significantly only from second-year students (F(5, 382) = 11.458, *p* < 0.001, η^2^ = 0.0982). However, formation graduates outperformed second-, third-, and fourth-year students, and undergraduate formation students scored significantly higher than all other groups (F(5, 382) = 11.458, *p* < 0.001, η^2^ = 0.0982). For the fourth sub-factor, “communication and access,” first-year students again had significantly more positive attitudes than second-, third-, and fourth-year students (F(5, 382) = 10.256, *p* < 0.001, η^2^ = 0.1624). Both undergraduate and graduate formation students scored significantly higher than second-, third-, and fourth-year students as well (F(5, 382) = 10.256, *p* < 0.001, η^2^ = 0.1624).

In the final sub-factor, “comparison of distance and face-to-face education,” first-year students reported more favorable attitudes than second-year students (F(5, 382) = 6.658, *p* < 0.001, η^2^ = 0.1578). Moreover, undergraduate formation students had significantly higher mean scores than first-, second-, and fourth-year students as well as formation graduates **(**F(5, 382) = 6.658, *p* < 0.001, η^2^ = 0.1578). Taken together, these findings indicate that students in the early stages of their academic journey and those participating in teacher formation programs tend to develop more positive attitudes towards distance education.

### Findings on pre-service teachers’ motivation towards distance learning

In the study, the mean and standard deviation values of students’ motivation levels for distance learning were analyzed. The mean score of students’ motivation was 𝑥̅ = 3.30 (SD = 0.368), indicating that their motivation was slightly above the mid-level. This suggests that students generally show a positive trend in their motivation towards distance learning, though not at very high levels.

To examine whether motivation levels varied by sex, an independent samples t-test was conducted. The results showed that female students (𝑥̅ = 3.30, SD = 0.368) and male students (𝑥̅ = 3.32, SD = 0.369) reported very similar motivation levels, and the difference was not statistically significant (t(386) = −0.601, *p* = 0.548).

The status of students’ motivation according to their classes is presented in Table 2.

**Table 2:** ANOVA findings to determine the motivations of pre-service teachers’ towards distance learning according to their grade levels.

Variables	Grade levels	N	$\bar{X}$	Ss	F	*p*	Eta squared (𝜂^2^)
Motivation	1	68	3.40	0.317	6.585	0.000	0.0793
	2	102	3.18	0.294			
	3	23	3.22	0.427			
	4	71	3.28	0.411			
	Formation (Graduate)	47	3.31	0.286			
	Formation (Undergraduate)	77	3.46	0.415			
	Total	388	3.30	0.368			

As shown in Table 2 the students’ mean motivation was highest in Formation (Undergraduate; 𝑥̅=3.46) and lowest in 2nd grade (𝑥̅=3.18). The total mean was (𝑥̅=3.30). One-way analysis of variance was performed to determine whether students’ motivation varied according to their grades. This result shows that there are some differences in motivation levels between different grade levels and programs.

Results showed that that the motivation levels of the students differed statistically according to the classes (*F* = 6.585; *p* < 0.05; η^2^ = 0.0793). In order to determine the difference in students’ motivation levels according to grades, LSD test was performed due to the homogeneous distribution of the groups. According to the results of the analysis, there is a significant difference be-tween 1st and 2nd grade in favor of 1st grade and between formation (undergraduate) and 2nd grade in favor of formation (undergraduate). 1st year students are usually new to the university and start with a higher level of excitement and motivation about the education-al process. During this period, students have a new learning experience and may have high expectations for the future. Similarly, the high motivation level of formation (under-graduate) students may be related to the professional preparation and practical training opportunities offered by the program. Such programs may allow students to make a more concrete connection to their career goals, which may increase their motivation.

### Findings on the relationship and predictive power of attitudes towards distance learning on student motivation

In the study, correlation analysis was conducted to determine whether there was a significant relationship between students’ attitudes and motivation towards distance learning. Simple regression analysis was conducted to determine to what extent students’ attitudes predicted their motivation. The findings are presented in Table 3.

**Table 3:** Simple regression analysis results regarding the predictive power of students’ attitudes on motivation.

Independent variable	Dependent variable	B	R	R^2^	SE	*β*	T	*p*
Attitude	Motivation	0.343	0.652	0.426	0.064	0.652	16.880	0.000

As presented in Table 3, there is a significant positive relationship between students’ attitudes and motivation above the medium level (*r* = 0.652, *p* < 0.05). A simple linear regression analysis was conducted to determine the extent to which students’ attitudes predicted their motivation. According to the analysis, a significant relationship was observed between attitude and motivation (R = 0.652; R2 = 0.425) and attitude was found to be a significant predictor of motivation (F(1–386) = 284.922, *p* < 0.01). According to these findings, attitude predicts motivation positively and significantly and ex-plains approximately 43% of motivation. This finding reveals that attitudes are an important factor shaping students’ motivation levels, indicating that attitude-oriented interventions in education can be effective in increasing motivation.

## Discussion, conclusion and future research

The pre-service teachers’ attitudes towards distance learning are slightly above the mid-level, suggesting a moderately positive psychological disposition toward digital learning environments. This result is in line with the literature (Araz et al., 2023; Küloğlu and Yıldız, 2022; Cansu, 2021; Halitoğlu, 2021; Marjerison et al., 2020; Ateş and Altun, 2008; Knowles and Kerkman, 2007; İlter et al., 2005; Brinkerhoff and Koroghlanian, 2005). However, other studies have reported lower levels of positive attitudes, which may reflect differences in students’ digital self-efficacy and psychological readiness (Kaban, 2021; Yakar and Yakar, 2021; Hacıömeroğlu and Elmalı-Erdem, 2021; Karatepe et al., 2020; Barış, 2015). In his study, Yağan (2021) found that students’ attitudes to-wards distance learning were at a low level. This finding may reflect increased learning anxiety and low perceived digital competence. These factors can reduce students’ psychological engagement with online education. One possible explanation for these findings lies in students’ levels of self-efficacy and cognitive adaptability. According to Bandura (1997), self-efficacy reflects students’ beliefs in their ability to successfully handle the challenges of online learning environments. Students with higher self-efficacy may feel more capable of managing technical difficulties, maintaining focus, and sustaining their academic engagement (Zimmerman, 2000; Artino, 2008). Similarly, students with stronger cognitive adaptability are better able to adjust their learning strategies when faced with unexpected changes, such as shifts to online platforms or sudden disruptions due to emergencies like natural disasters (Martin et al., 2013; Ployhart and Bliese, 2006). He also found that students prefer face-to-face education to distance learning, that the efficiency in distance learning is low, that this learning causes laziness and prevents socialization. He stated that especially the belief that applied courses cannot be given by distance learning is dominant.

Kaya et al. (2022) concluded in their study that students find face-to-face education more effective, distance learning is not interesting and qualified results cannot be obtained from distance learning applications. Similarly, Kışla (2015) reported that participants’ attitudes were ambivalent, likely due to digital stress and insufficient access to technological resources at home. Eliminating system difficulties and technical problems may improve students’ attitudes toward distance learning. Kaban (2021) also found that having a computer and stable internet connection, as well as regular participation in virtual classes, positively influences attitudes. Furthermore, the exclusive use of distance learning for all courses might contribute to negative attitudes; thus, blended learning approaches—by mitigating cognitive overload and fostering interpersonal interaction—could provide an effective solution (Özkul and Aydın, 2012).

The findings regarding pre-service teachers’ attitudes and motivation levels are significant from a psychological perspective, as they indicate that even moderately positive attitudes can enhance intrinsic motivation and learning engagement. First of all, pre-service teachers’ generally positive attitudes towards distance learning are promising for the broader adoption of this model, particularly in contexts where digital education is expanding as a complement to face-to-face instruction. Increased access to educational resources is known to support student engagement. The current study provides empirical evidence that this access also leads to measurable motivational gains for pre-service teachers. In our sample, students who reported having stable internet connections and regular participation in online courses demonstrated significantly higher motivation scores. This suggests that access is not merely a logistical facilitator. It also functions as a psychological enabler by reducing anxiety, enhancing perceived competence, and supporting academic continuity. Moreover, this finding aligns with previous research indicating that technological accessibility is closely linked to students’ psychological readiness and sustained engagement in digital learning environments (e.g., Kaban, 2021; Yakar and Yakar, 2021). At the same time, the motivational benefits of access may vary depending on contextual factors such as socioeconomic status, institutional support, and regional infrastructure. Therefore, improved access should not only be interpreted as a logistical advantage but as a critical motivational factor within digital learning environments. From a practical perspective, these results highlight the importance of ensuring equal access to digital resources, as doing so may reduce disparities in motivation and academic achievement across diverse student populations. The Fuzzy Cognitive Maps (FCM) model pro-posed in Lepore (2024) offers an innovative approach to assessing the impact of digital tools in distance education by analyzing students’ motivation, engagement and cognitive processes in a multidimensional framework. The data generated by this model help explain how digital learning environments affect students’ cognitive and emotional states, particularly under pandemic conditions. These results support the findings of the present study. Similarly, in this study, positive attitudes towards distance education were found to significantly increase students’ motivation. In addition, Lepore (2024) stated that the integration of interaction-oriented digital tools that provide personalized feedback to students can more effectively support motivation and engagement in distance education. Therefore, when these two studies are evaluated together, it can be concluded that comprehensive models that address cognitive, emotional and social factors together should be developed for the sustainable success of distance education.

Sex does not create any significant difference in students’ attitudes. Although no significant gender difference was found in attitudes towards distance learning, this result should be interpreted in light of the evolving nature of digital learning environments. One possible explanation is that increased exposure to technology has reduced gender disparities in digital literacy and confidence. Furthermore, the normalization of online learning during global crises may have contributed to more equalized experiences across genders. However, some previous studies have noted gender-based variations—such as males exhibiting more task-oriented motivation or females showing stronger self-regulation skills—suggesting that contextual variables like prior experience, technological accessibility, and learning strategies may act as moderators. Thus, future research should further investigate these nuances through interaction analyses or qualitative inquiry. As in this study, other research indicates that the sex variable does not affect attitudes toward distance learning (Ateş and Altun, 2008; Akgün, 2015; Yakar and Yakar, 2021; Güney and Mete, 2022). Nonetheless, some studies have found higher positive attitudes among males, which may be related to gender differences in digital self-efficacy (Horzum et al., 2012; Yenilmez et al., 2017). The attitude levels of the students differ significantly according to their grades, with formation students and first-year undergraduates exhibiting significantly more positive attitudes towards distance learning. Formation students study in different departments and classroom settings with an interdisciplinary perspective. This experience may have positively influenced their attitudes toward distance learning. The attitude levels of students differ significantly according to their grades. Formation students and first-year undergraduates—who may have lower cognitive load and fewer prior negative experiences with online education—tend to have higher positive attitudes, possibly reflecting developmental differences in psychological adaptability and self-regulation (Hacıömeroğlu and Elmalı-Erdem, 2021; Bayram et al., 2019; Ateş and Altun, 2008). The fact that sex does not have a significant effect on attitudes may indicate that distance learning processes are more inclusive. This suggests that educational environments may be becoming more egalitarian, at least in terms of digital literacy and motivational readiness, enabling individuals to have more effective learning experiences regardless of sex. Branchetti et al. (2021) emphasize how distance learning has transformed traditional teaching methods and redefined teaching boundaries. This transformation has enabled teachers and students to approach the distance learning process in a more flexible and innovative way. Fiore et al. (2022) also focus on distance learning and multimedia education, noting that it can positively affect students’ attitudes towards learning processes and increase interaction.

The motivation of pre-service teachers towards distance learning is slightly above the mid-level. From a psychological standpoint, this moderate motivation indicates partial engagement among students. There remains potential to enhance intrinsic factors such as autonomy and perceived competence (Ryan and Deci, 2020). In this context, self-efficacy also plays a crucial mediating role in shaping students’ motivation levels. Students who perceive themselves as competent and capable in managing online learning tasks are more likely to maintain higher levels of intrinsic motivation (Schunk and DiBenedetto, 2020; Lee et al., 2014). Cognitive adaptability also supports motivation by helping students manage technological uncertainties and adapt to changing online environments. This flexibility reduces frustration and academic disengagement (Toland and Carrigan, 2011; Haynie, 2005). Sex does not create any significant difference in students’ motivation. There are studies reaching similar results in the literature. Malinauskas and Pozeriene (2020) and Özüdoğru (2021) concluded that students’ online learning motivation scores were above average and sex did not make a significant difference on motivation. In the literature, there are also studies in which students’ motivation in distance learning is low and many different problems are experienced. Şimşek (2022) found that the student’s motivation deteriorated due to technical features and communication problems in the home environment where distance learning is applied. Yıldız (2016), in his study, mentioned the technical problems experienced by students, the lack of communication with teachers and the motivation problem arising from individual work.

It is stated that one of the biggest disadvantages of distance learning is the lack of social interaction (Aguilera-Hermida, 2020; Dutta and Smita, 2020). Reduced student–student and student–instructor interaction can lead to feelings of isolation and decreased aca-demic self-efficacy, ultimately diminishing motivation (Logan et al., 2017; Shea and Bidjerano, 2009). Moreover, the traditional lecture-note format may result in decreased engagement and a functional rather than stimulating learning experience, with increased educational load further intensifying these psychological challenges (Yazgan, 2022). These problems experienced in distance learning negatively affect students’ academic achievement along with motivation. Because Logan et al. (2017) stated that high levels of motivation of have a positive relationship with academic performance in distance learning environment. It is stated that online learning activities may not support students’ motivation and learning performance if they are not well designed and developed (Zhou and Zhang, 2023). Barba et al. (2016) stated that there is a positive relationship between motivation and participation in online environments. It is also stated that students with high motivation can interact more with the learning content (Çebi, 2023; Çebi and Güyer, 2020; Eom, 2018; Fanguy et al., 2018).

Capone and Lepore (2022) state that distance learning practices can increase motivation, but for this process to be successful, effective teaching methods and the use of technological technologies can be increased. A case study by Capone et al. (2022) shows the emergence of centralized “distance coursework” practices that allow students to enhance their learning experience by collaborating in courses that are spread far and wide. Bayındır (2021) stated that the most important motivational factor in online learning experiences is the duration of the course. From a psychological perspective, these findings underscore the importance of structured digital learning environments that foster self-regulation and provide social support (Schunk and DiBenedetto, 2020). He thinks that academic communication increases the motivation period and believes that digital teaching practices have important contributions to motivation. In this context, it is important to keep the duration of online courses short, to ensure active teacher-student communication in the course, and to use digital teaching tools and modules appropriate to the course content. The time spent in the distance learning environment and the frequency of participation in synchronized courses were found to be factors affecting students’ motivation for distance learning (Bertiz and Karoğlu, 2020).

Students’ motivation levels also differ significantly according to their grades. The higher motivation observed among formation students and first-year undergraduates may reflect their initial excitement and lower cognitive burden, while students in higher grades, who face more complex academic challenges, might experience decreased motivation. These findings suggest that curricula should evolve in line with students’ changing psychological needs. For instance, higher-grade courses could emphasize self-regulation skills and reduce cognitive overload (Sarıtaş and Barutçu, 2020; Baygeldi et al., 2021).

The observed differences in attitudes and motivation across grade levels may not only reflect first-year enthusiasm but could also be influenced by variations in academic workload, adaptation stress, and the development of self-regulation skills as students progress through their programs. Higher-grade students may experience greater cognitive demands, role conflicts, and external responsibilities, which may affect their psychological readiness for distance learning (Zimmerman, 2000; Schunk and DiBenedetto, 2020).

The emergence of motivation differences across grade levels highlights how pre-service teachers’ learning experiences evolve over time, likely influenced by increased academic pressure and cumulative cognitive load. Some studies have also examined the negative and unexpected consequences of distance learning. Hodges et al. (2020) state the negative effects of technological problems in the distance learning process on students’ learning experiences. Kuo and Belland (2016) examine the effects of the distance learning environment on distractions and lack of convenience.

Ferguson (2012) discusses how a lack of social interaction in distance learning con-tributes to social isolation and decreased motivation. Baker (2010) and Shea and Bidjerano (2009) further highlight that insufficient teacher support and low participation rates can negatively impact motivation. Overall, a significant positive relationship between pre-service teachers’ attitudes towards distance learning and their motivation has been consistently observed, with attitude explaining approximately 43% of the variance in motivation. This strong association may reflect the combined influence of students’ psychological resources, such as self-efficacy and cognitive adaptability. As students gain confidence in managing online learning tasks and adapting to new academic demands, their attitudes toward distance learning become more positive. This, in turn, enhances their motivation (Martin et al., 2013; Ryan and Deci, 2020). This indicates that as positive attitudes increase, so does motivation.

These findings are further supported by Self-Determination Theory (SDT; Ryan and Deci, 2020), which posits that motivation is sustained when individuals experience autonomy, competence, and relatedness. In the context of distance learning, positive attitudes may strengthen perceived autonomy and competence by fostering a sense of control over learning tasks and confidence in one’s digital abilities. When these psychological needs are fulfilled, students engage more voluntarily and persistently in online education. External learning requirements are then transformed into intrinsic motivation.

The results also have practical implications for teacher-training programs. Teacher education programs could integrate reflective workshops, digital pedagogy modules, and collaborative e-learning projects. These components may improve pre-service teachers’ attitudes toward technology-based instruction. Such interventions would not only strengthen positive perceptions of distance education but also promote intrinsic motivation and engagement, equipping future educators with the psychological readiness to integrate digital tools effectively in their own classrooms.

A key finding of the current study is the statistically significant and positive predictive relationship between pre-service teachers’ attitudes and their motivation towards distance learning. However, this relationship—while moderately strong—should not be interpreted as strictly unidirectional or purely causal. The use of a simple linear regression model implies a direct, one-way influence of attitude on motivation. However, such models may oversimplify complex psychological relationships.

In fact, the relationship between attitude and motivation may be bidirectional. While positive attitudes can enhance motivation, heightened motivation may also lead to more favorable attitudes toward online learning. Because the present study employed a cross-sectional design, it cannot determine causal direction. Future longitudinal or experimental studies could further clarify this reciprocal dynamic and explore how these constructs reinforce one another over time.

Motivation is influenced by various interacting factors, including self-efficacy, prior experience with online education, perceived autonomy, and emotional regulation. These variables may act as mediators or moderators in the relationship between attitudes and motivation. For example, students with high self-efficacy may experience greater motivational gains even when their attitudes are only moderately positive. Conversely, students with low digital competence or high cognitive overload might report low motivation regardless of attitude scores.

Therefore, while the present study provides empirical evidence for a predictive link, the findings should be interpreted within the methodological limitations of the linear framework. Future research could extend this model by testing potential mediating or moderating variables—such as self-efficacy, cognitive adaptability, or emotional regulation—that may explain or influence the relationship between attitude and motivation. Such extensions would provide a more comprehensive understanding of the psychological mechanisms that influence pre-service teachers’ engagement in distance learning. They would also strengthen the theoretical alignment between conceptual and empirical components of the model. While this study employed simple linear regression to explore the predictive relationship between attitudes and motivation, future research could benefit from more advanced statistical techniques such as mediation, moderation, or structural equation modeling (SEM). These methods would enable a deeper understanding of the complex psychological mechanisms underlying students’ attitudes and motivation. For instance, self-efficacy or digital literacy may function as mediators, while gender, grade level, or prior online learning experience could act as moderators. Using such models could uncover indirect effects and interaction patterns, providing a more comprehensive psychological framework for analyzing distance learning dynamics.

While several instructional strategies (e.g., gamification, mentoring, analytics) are discussed as potential enhancements, these suggestions are offered as general recommendations informed by the broader literature, rather than directly derived from the current dataset.

This study has several limitations that should be considered when interpreting the findings. First, the sample was drawn from a single public university, which restricts the generalizability of the results to other cultural and institutional contexts. Additionally, technological access variables (e.g., device ownership, internet quality) were not directly measured, which may have influenced students’ attitudes and motivation. Data were collected during one academic semester (Fall 2023–2024), providing a cross-sectional view that may not reflect long-term changes in students’ perceptions. Although emotional and psychological constructs such as resilience, anxiety, and trauma are highly relevant in distance learning contexts, they were not empirically measured in this study. The study was limited to correlation and regression analyses, which do not capture potential mediating or moderating relationships among variables. The regression model also reflects a one-directional relationship, which may oversimplify the multidimensional nature of motivation. Future studies may benefit from more diverse samples, longitudinal designs, and advanced analyses such as mediation, moderation, or structural equation modeling to explore causal mechanisms more comprehensively.

Based on these findings and limitations, several recommendations for future research and practice emerge. Enhancing communication and socialization through interactive digital tools, virtual group work, and online discussion platforms may improve both attitudes and motivation. In addition, targeted interventions aimed at strengthening students’ self-efficacy beliefs and developing cognitive adaptability skills could provide further benefits. Such interventions may include structured digital literacy training, adaptive problem-solving workshops, and resilience-building programs that prepare students for the dynamic nature of distance education environments (Bozkurt and Sharma, 2020; Schunk and DiBenedetto, 2020). Additionally, limiting analyses to specific faculties or class levels could provide deeper insights into the psychological underpinnings of these constructs. Employing qualitative methods such as phenomenology and case studies would offer a richer, multi-dimensional perspective on the cognitive and emotional experiences of learners in distance education environments.

The current study is limited to students from one public university. Future research should include diverse cultural and institutional contexts to enhance the generalizability of the findings. In future research, including participants from different regions, cultural backgrounds and from various types of universities (public, foundation, international) may increase the generalizability of the findings.

Although psychological factors such as trauma, anxiety, and resilience are highly relevant in emergency-based distance learning contexts, these variables were not directly measured in this study. Future research may benefit from including these constructs to provide a more comprehensive understanding of students’ psychological adjustment during natural disasters.

In the study, data were collected in a specific period. In the future, longitudinal studies can be conducted to examine the effects of distance education on attitudes and motivation in the context of changing dynamics over time. This may reveal the reasons for changes in attitudes and motivation and their long-term effects.

Factors such as level of technological access and psychological state were not assessed in the study. Future studies could examine the effects of participants’ access to digital tools, technological literacy and psychological health on attitudes and motivation to-wards distance education.

This study targeted a general student population. Comparing the attitudes and motivations of students in different academic disciplines towards distance education may help to reveal interdisciplinary differences.

Based on the effects of lack of interaction between students and social isolation on motivation, future research could focus on strategies that increase interaction (e.g., virtual group work, online discussion platforms). The effects of these strategies can be tested with experimental methods. Future research may consider employing more complex methodological approaches, such as experimental or longitudinal designs, or structural equation modeling, to further investigate the causal mechanisms between attitudes towards distance education and motivation. While the current study adopts a predictive and correlational framework, it provides a foundational basis for understanding the psychological dimensions of students’ distance learning experiences. The findings of this research may serve as a reference point for designing targeted interventions and informing future empirical models aimed at enhancing student engagement and motivation in digital learning environments.

Future research would benefit from employing more robust statistical techniques such as hierarchical regression, path analysis, or structural equation modeling (SEM). These approaches can account for interaction effects and uncover indirect pathways, thereby offering a more comprehensive psychological explanation of motivation in digital learning environments. Such enhancements would strengthen the theoretical and practical implications of findings and help guide more tailored interventions in distance education.

Mixed methods can be used to examine attitudes and motivation towards distance learning. A combination of both quantitative and qualitative data can provide more in-depth analysis and a better understanding of participants’ experiences. Studies examining the long- term effects of distance learning processes can provide an opportunity to track changes in attitudes and motivation over time. Comparative studies could be con-ducted to investigate how attitudes and motivation towards distance learning change across different disciplines and levels of education. This could help to better understand the overall effects of distance learning.

Although the concepts of resilience and cognitive adaptability are discussed in the theoretical framework, they were not directly measured in this study. This represents a limitation in aligning the theoretical constructs with empirical analysis. Future research should consider incorporating validated measurement tools for resilience and adaptability in order to strengthen the theoretical coherence and provide a more comprehensive psychological profile of students in distance learning contexts.

Use e-learning analytics to improve student engagement and success. Data analytics can also be used to track student behavior and intervene according to needs. Implementing gamification elements in distance learning environments can increase motivation. New methodologies can be developed to encourage student engagement by adding game elements. Interactive activities such as online group projects and discussion forums can be designed to increase students’ social interaction. This can increase students’ motivation by reducing their sense of social isolation. Mentoring programs and guidance services can be created to provide individual support to students. Providing personalized learning experiences based on students’ needs can increase motivation. Educational institutions should provide a strong technological infrastructure to support students’ distance learning experiences. Providing reliable internet connections and accessible educational materials can increase engagement and motivation.

The positive relationship between attitude and motivation may emphasize the importance of applying various strategies to improve pre-service teachers’ attitudes in educational processes. Identifying the factors that positively affect attitudes and conducting studies in this direction can contribute to making teaching processes more effective.

Finally, future research could explore the effects of social isolation on motivation and test strategies designed to increase interaction and engagement, such as gamification, e-learning analytics, and mentoring programs. Developing comprehensive models that integrate cognitive, emotional, and social dimensions will be essential for designing effective, psychologically-informed distance learning environments.

Unlike pandemic-era studies focusing primarily on technological readiness, this research uniquely captures pre-service teachers’ psychological adjustment and motivational recovery in a post-disaster educational context, offering context-specific insights for educational psychology.

In conclusion, the findings suggest that pre-service teachers develop a positive perspective towards educational processes when engaging in distance learning. Supporting these positive attitudes through targeted programs and strategies can enrich the learning experiences of both teachers and students. Ultimately, dynamic and interactive learning environments are needed to promote collaboration and continuous engagement. Approaches such as gamification, mentoring, and e-learning analytics appear essential for sustaining this transformation in education.

## Data Availability

The original contributions presented in the study are included in the article/supplementary material, further inquiries can be directed to the corresponding author.
